# Questioning the Causal Link between Maternal Smoking during Pregnancy and Offspring Use of Psychotropic Medication: A Sibling Design Analysis

**DOI:** 10.1371/journal.pone.0063420

**Published:** 2013-05-08

**Authors:** Lovisa Söderström, Raquel Perez-Vicente, Sol Juárez, Juan Merlo

**Affiliations:** 1 Unit for Social Epidemiology, Department of Clinical Sciences, Faculty of Medicine, Lund University, Malmö, Sweden; 2 Centre for Economic Demography, Lund University, Malmö, Sweden; National Institutes of Health - National Institute of Child Health and Human Development, United States of America

## Abstract

A recent population-based, longitudinal study from Finland observed a dose-response association between smoking during pregnancy (SDP) and use of psychotropic medications in exposed children and young adults. However, this association may be confounded by unmeasured familial characteristics related to both SDP and offspring mental health. Consequently, we aim to investigate the effect of SDP by means of a sibling design that to some extent allows controlling for unknown environmental and genetic confounders. Using the Swedish Medical Birth Register (1987–1993), which was linked to the Swedish Prescribed Drugs Register (July 2005–December 2008), we investigated 579,543 children and among them 39, 007 were discordant for use of psychotropic medication and 4,021 siblings discordant for both use of psychotropic medication and for smoking exposure. Replicating the Finnish study using traditional logistic regression methods we found an association between exposure to ≥10 cigarettes per day during pregnancy and psychotropic drug use (odds ratio = 1.61, 95% confidence interval 1.56, 1.66). Similar in size to the association reported from Finland (odds ratio = 1.63; 95% confidence interval 1.53, 1.74). However, in the adjusted sibling analysis using conditional logistic regression, the association was considerably reduced (odds ratio 1.22; 95% confidence interval 1.08, 1.38). Preventing smoking is of major public health importance. However, SDP *per se* appears to have less influence on offspring psychotropic drug use than previously suggested.

## Introduction

Several studies have reported an association between maternal smoking during pregnancy (SDP) and offspring psychological disorders. These include mainly externalizing behavioral disorders, such as attention deficit hyperactivity disorder (ADHD), conduct disorder, and oppositional defiant disorder, but also internalizing psychopathology (i.e., depression and anxiety disorders) [1], [2], [3], [4]. Ekblad et al have recently observed a dose-dependent association between maternal SDP and offspring use of psychotropic medication during childhood and up to young adulthood [5]. Their hypothesis was that prenatal smoking exposure interferes with the development of the fetal brain and, thus, increases psychiatric morbidity, leading to increased risk for use of psychotropic medications. Compared to children unexposed to SDP, the authors found odds ratios (ORs) of 1.36 and 1.63, respectively, for exposure to less than, and more than, 10 cigarettes per day. Their analysis was adjusted for sex, maternal age, obstetric characteristics, as well as psychiatric diagnosis of the mother.

However, almost simultaneously with Ekblad and coauthors [5], another study by Lavigne et al questioned the role of maternal SDP as a risk factor for psychopathology in young children [6]. The analysis by Lavigne et al controlled for a more detailed set of potential confounders, such as socioeconomic status, life stress, family conflict, maternal depression, maternal scaffolding skills, mother–child attachment, and other variables that were not available in the large record linkage databases used by Ekblad et al [5].

Investigating causality through observational studies is problematic and the study of long-term causal effects of prenatal smoking exposure presents specific difficulties [7], [8], [9]. In fact, psychological problems of the mother, like depression and neuroticism, as well as socioeconomic disadvantage may be common causes of both maternal SDP [10], [11] and offspring psychiatric morbidity. Therefore, it is possible that the results obtained by Ekblad et al [5] are due to residual confounding through environmental circumstances in these families or through an inherited risk for psychiatric morbidity.

Recently, in an attempt to reduce residual confounding, several studies applied a design comparing siblings differently exposed to maternal SDP [12], [13], [14], [15], [16], [17]. These studies suggest that associations between maternal SDP and childhood outcomes, such as externalizing behavior, ADHD, antisocial behavior, hyperkinetic disorder, criminality, and poor academic achievement, are largely due to familial confounding [12], [13], [14], [15], [16], [17]. Other studies have tried to reduce residual confounding by distinguishing between maternal and paternal SDP because paternal SDP does not cause direct intrauterine exposure to tobacco. Using this epidemiological design, a small study by Nomura, Marks, and Halperin [18] found that only maternal SDP was associated with ADHD symptoms. However, Langley, Heron, Smith, and Thapar [19] observed an association between both maternal and paternal smoking and ADHD symptoms, suggesting that the association between maternal SDP and psychiatric outcomes may be due to genetic or household-level confounding rather than to the causal intrauterine effect of SDP.

If the association between maternal SDP and psychiatric morbidity in the offspring was causal, this would further support the evidence that maternal smoking in this critical period of life puts the fetus at risk. If not, prevention should also be focused on improving those socioeconomic and psychosocial circumstances of the mother that are common causes of both SDP and offspring mental ill health.

Therefore, applying the sibling design, we aimed to revisit the association between maternal SDP and increased psychotropic drug use in children and young adults recently reported by Ekblad et al [5]. The sibling design is suited to study causal associations since it approximates a counterfactual situation of exposure [20]. This design assesses the impact of maternal SDP on the offspring use of psychotropic medication in individuals who are genetically related and share a similar social environment [21], [22]. Sibling designs are, therefore, able of adjusting for unmeasured and even unknown factors that are a common cause of both maternal SDP and offspring use of psychotropic medication [23].

For doing these sibling analyses we applied conditional logistic regression (CLR). CLR is suitable for matched case control studies. In our case, we can understand the sibling analysis as a matched case-control study where one of the siblings is a “case” (i.e., use psychotropic drugs) and the other sibling the “control” (i.e., non-use of psychotropic drugs), and the matching variable is the mother. In the CLR analyses the estimations are obtained in siblings with discordant outcome (i.e., cases and controls) and exposure (smoking vs non-smoking). The association between SDP and psychotropic drugs is, by design, adjusted for the matching variable (mother). Therefore, the CLR accounts for the correlation of the information between the case and control siblings.

However, the similarity between siblings and, thereby, the capacity of the sibling design for confounding adjustment should not be exaggerated [22], [24]. From this perspective it is necessary to control for variables that change between pregnancies and that may be a common cause of both maternal SDP and offspring use of psychotropic medication.

On this background, we analyzed outpatient psychotropic drug use between 2005 and 2008 in children and adolescents born in Sweden between 1987 and 1993.

## Materials and Methods

### Study Population and Ethics Statement

The Swedish Medical Birth Register collects standardized information on the antenatal care, delivery, and medical examination of newborn babies. It includes about 98.6% of all pregnancies in Sweden that culminate in delivery [25], [26]. The National Board of Health and Welfare, in coordination with Statistics Sweden, links the Swedish Medical Birth Register to a number of other national databases: the Swedish Prescribed Drugs Register, the National Mortality Register, the Emigration Register, the National Inpatient Register, and the Income and Asset Register. This record linkage was performed by the Swedish authorities using a unique personal identification number given to each person residing in Sweden. However, in the data we analyzed, the identification numbers were replaced with arbitrary numbers to safeguard the anonymity of the subjects.

The construction of the record linkage database used in our study was approved by The Regional Ethical Review Board in Southern Sweden, The National Board of Health and Welfare and Statistics Sweden. Lund Universidad signed a contract of confidentially with the Swedish Authorities. Active informed consent was waived as a requirement for the construction of the database.

We identified all 811,599 children born between January 1^st^, 1987, and December 31^st^, 1993, recorded in the Swedish Medical Birth Register. We excluded every non-singleton child (n = 19,162). Measurement of psychotropic drug use using administrative registries reflects both access to healthcare and the presence of psychological disorder, which may originate information bias when studying the effect of smoking. Therefore, we also excluded every child with an immigrant parent (n = 157,856) because children of immigrants have been reported to use less psychotropic medication in relation to their needs (see elsewhere for a more detailed discussion [22]).

**Figure 1:**
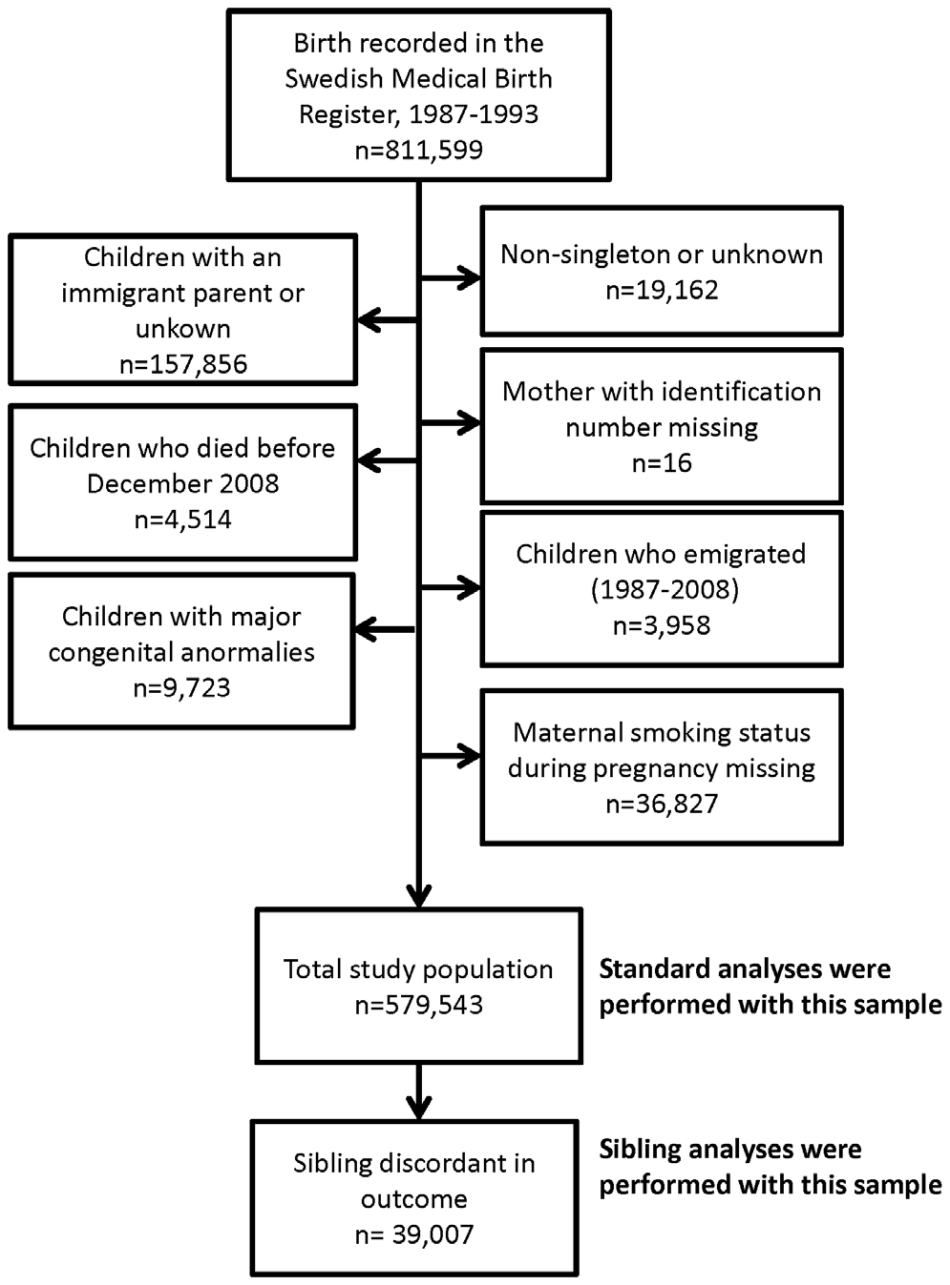
Flow diagram indicating the selection criteria and the number of individuals included in the analyses.

We furthermore excluded children whose mothers’ identification number was missing (n = 16), children who had died (n = 4,514) or emigrated (n = 3,958) before December 31^st^, 2008, and children with missing information on maternal SDP (n = 36,827). To further increase the homogeneity of our study, we also excluded children with major congenital abnormalities (n = 9,723). The final study population for performing traditional analyses consisted of 579,543 subjects. The sibling analyses were done on a sample of 39,007 siblings with discordant outcome (Figure 1). Out of these, 4,021 were siblings with contrast of exposure and outcome.

The individuals were between 11 and 21 years of age when we gathered information about their use of psychotropic medication.

### Assessment of Outcome Variable

The Swedish Prescribed Drug Register contains all dispensed medication prescribed in outpatient settings in Sweden since July 2005. It does not contain over the counter medication or medication given in hospitals and nursing homes [27]. From this registry, we identified six different categories of psychotropic medication, according to the Anatomical Therapeutic Chemical (ATC) classification system: antipsychotics (N05A), anxiolytics (N05B), hypnotics and sedatives (N05C), antidepressants (N06A), psychostimulants (N06B), and medication used in addictive disorders (N07B). We defined the outcome as at least one dispensed prescription (“yes”/“no”) of one of these medications during the period from July 1^st^, 2005, to December 31^st^, 2008.

Because we use psychotropic medication as a proxy of psychiatric disorders and the therapeutic profiles of the different medication groups overlap each other (i.e., different psychiatric disorders can be treated with the same drug group), we study psychotropic medication as a group.

### Assessment of Maternal Smoking during Pregnancy

From the Swedish Medical Birth Register, we obtained information on self-reported SDP. This information was retrieved at the first antenatal care visit (i.e., between gestational weeks 8 and 12) [28]. Smoking during pregnancy was categorized as no smoking, 1–9 cigarettes per day, and ≥10 cigarettes per day.

### Assessment of Other Variables

From the Swedish Medical Birth Register, we collected information on parental relationship status, parity, birth order, Apgar score, the mother’s age at delivery, gestational age (GA) in weeks, birth weight, and birth weight adjusted for GA. Information on parents’ relationship status is reported by the mother at the first antenatal care visit. Apgar score is rated on a scale of 1–10 at 1, 5, and 10 minutes after birth. A score of 10 indicates a delivery without fetal distress. In the analyses, we used the 5-minute Apgar score. The variable “birth weight adjusted for GA” is categorized in the Swedish Medical Birth Register as small for gestational age (SGA), appropriate for gestational age (AGA), and large for gestational age (LGA). Small for gestational age and LGA are defined as a birth weight below or above 2 standard deviations (SDs) from the average birth weight for those born at that GA in Sweden.

From the Income and Asset Register, we obtained information on whether the parents were receiving social welfare the year before the birth of the child. We also obtained information on the income of the parents the year before and after the birth of the child. We created a combined variable categorized into four groups using income tertiles and adding a fourth category for parents receiving social allowance. Using the National Inpatient Register, we collected data on maternal history of psychiatric diagnosis if the mother had at least one diagnosis coded 290–319 or F00-F99, according to the International Classification of Diseases and Related Health Problems (ICD), 9th and 10^th^ revisions, respectively, during the period 1973–2005. For most variables with continuous and ordinal data, we created categories, as shown in Tables 1 and 2.

**Table 1:** Characteristics of the children and adolescents born in Sweden between 1987 and 1993 and living in Sweden between July 2005 and 2008 by maternal smoking during pregnancy (SDP) and offspring use of psychotropic medication.

	Full Sample			Siblings Sample		
Characteristics	Number of individuals	MaternalSDP (%)	Psychotropicdrug use (%)	Number of individuals	MaternalSDP (%)	Psychotropic drug use (%)
Study Population	579,543 (100.0)	24.6	7.2	39,007 (100.0)	28.6	47.2
Maternal smoking						
No smoking	437,164 (75.4)		6.4	27,867 (71.4)		46.6
1–9 cigarettes/day	88,970 (15.5)		9.1	6,499 (16.7)		48.8
>9 cigarettes/day	53,409 (9.2)		10.8	4,641 (11.9)		48.2
Sex						
Female	297,239 (51.3)	24.6	8.6	20,437 (52.4)	28.9	52.8
Male	282,304 (48.7)	24.5	5.9	18,570 (47.6)	28.3	41.0
Birth year (age in yrs)						
1987 (17–21)	75,023 (13.0)	28.0	10.6	5,658 (14.5)	31.2	59.0
1988 (16–20)	79,779 (13.8)	26.6	9.3	5,368 (13.8)	31.3	56.2
1989 (15–19)	81,951 (14.1)	25.7	8.5	6,315 (16.2)	28.9	52.6
1990 (14–18)	85,435 (14.7)	24.9	7.3	6,519 (16.7)	27.3	47.9
1991 (13–17)	88,848 (15.3)	24.0	6.0	5,994 (15.4)	27.5	41.8
1992 (12–16)	86,946 (15.0)	23.0	5.1	4,858 (12.5)	27.3	25.4
1993 (11–15)	81,561 (14.1)	20.4	4.2	4,295 (11.0)	26.0	31.9
Maternal age at pregnancy (years)						
<20	14,195 (2.5)	44.8	12.2	1,132 (2.9)	47.4	54.4
20–29	353,379 (61.0)	25.3	7.2	26,358 (67.6)	30.2	48.7
30–39	202,046 (34.9)	22.0	6.7	11,210 (28.7)	23.0	42.9
>39	9,923 (1.7)	22.1	8.4	307 (0.8)	19.9	40.4
Parity						
1	130,271 (22.5)	26.4	6.9	ND	ND	ND
2	268,400 (46.3)	22.6	7,0	21,332 (54.69)	26.2	50.0
3	129,239 (22.3)	24.5	7.4	11,783 (30.2)	29.3	43.9
>4	51,633 (8.9)	30.3	8.6	5,892 (15.1)	35.7	43.4
Birth order						
1	241,969 (41.8)	24.4	7.3	13,199 (33.8)	28.0	58.6
2	210,439 (36.3)	23.3	6.9	16,380 (42.0)	26.7	43.4
3	93,948 (16.2)	25.8	7.1	6,840 (17.5)	31.0	36.7
>4	33,187 (5.7)	30.8	8.4	2,588 (6.6)	37.2	40.1
Motheŕs psychiatric diagnosis						
No	553,862 (95.6)	23.6	6.9	37,293 (95.6)	27.9	47.0
Yes	25,681 (4.4)	45.5	14.4	1,714 (4,4)	43.3	50.4
Parents living together						
Yes	523,871 (90.4)	23.4	7.1	35,363 (90.7)	52.2	47.2
No	23,253 (4.0)	50.0	11.8	1,562 (4.0)	27.5	51.5
Missing	32,419 (5.6)	24.7	6.2	2,082 (5.3)	28.2	42.8
Income						
Highest	193,134 (33.3)	16.3	5.0	10,891 (27.9)	18.7	39.0
Middle	196,852 (34.0)	22.2	6.9	12,787 (32.8)	24.4	48.4
Lowest	142,679 (24.6)	28.4	8.4	10,120 (25.9)	29.6	53.5
Social allowance	46,863 (8.1)	57.3	13.9	5,206 (13.4)	57.3	48.9
Missing	15 (0.0)	26.7	13.3	3 (0.0)	33.3	33.3

**Table 2:** Obstetrics characteristics of the total study population of children and adolescents born in Sweden between 1987 and 1993 and living in Sweden between July 2005 and 2008 by maternal smoking during pregnancy (SDP) and offspring use of psychotropic medication.

	Full Sample			Siblings Sample		
Characteristics	Number of individuals (%)	Maternal SDP (%)	Psychotropic drug use (%)	Number of individuals (%)	Maternal SDP (%)	Psychotropic drug use (%)
GA, weeks						
<28	407 (0.1)	33.7	13.3	39 (0.1)	46.2	55.7
28–31	2,028 (0.4)	33.5	10.6	127 (0.3)	36.2	55.9
32–36	24,385 (4.2)	29.6	8.5	1,787 (4.6)	35.8	48.9
34–41	511,055 (88.2)	24.4	7.1	34,403 (88.2)	28.4	46.9
>41	41,347 (7.1)	23.1	7.2	2,622 (6.7)	25.3	49.0
Missing	321 (0.1)	39.6	10.0	29 (0.1	48.3	62.1
Birth weight, gr						
<2000	4,786 (0.8)	36.7	10.3	319 (0.8)	42.3	54.9
2000–2999	68,556 (11.8)	37.8	8.5	4,762 (12.2)	45.2	50.7
3000–3999	392,377 (67.7)	24.7	7.1	26,265 (67.3)	28.6	47.4
>3999	112,958 (19.5)	15.5	6.5	7,600 (19.5)	17.5	43.8
Missing	866 (0.2)	27.9	11.8	61 (0.2)	36.1	60.7
Birth weight adjusted for GA						
SGA	14,012 (2.4)	43.9	9.6	909 (2.3)	49.2	54.2
AGA	545,117 (94.1)	24.5	7.1	36,640 (93.9)	28.6	47.1
LGA	19,597 (3.4)	13.6	7.4	1,398 (3.6)	14.0	44.6
Missing	817 (0.1)	29.6	9.9	60 (0.2)	40.0	61.7
5- Minutes Apgar score						
0–3	1,255 (0.2)	24.1	10.4	84 (0,2)	34.5	67.9
4–6	3,297 (0.6)	27.3	10.1	225 (0,6)	32.9	57.8
7–10	566,571 (97.8)	24.5	7.2	38092 (97,7)	28.5	47.0
Missing	8,420 (1.5)	26.4	8.0	606 (1,6)	31.0	49.5

AGA = appropriate for gestational age; GA = gestational age; LGA = large for gestational age; SGA = small for gestational age.

### Statistical Methods

We analyzed the association between maternal SDP and use of psychotropic medication in a series of steps. Firstly (*model a*), we performed a traditional simple logistic regression analysis. Thereafter (*model b*), we replicated the study by Ekblad et al [5], by adjusting for similar variables as they did (i.e., sex, GA, birth weight, 5-minute Apgar score, maternal age, parity and maternal psychiatric diagnosis) in a traditional multiple logistic regression analysis. The outcome of our analysis was very similar to the outcome used by Ekblad et al [5].

Since we did not fully agree with the variables that Ekblad et al [5] used in their adjusted analysis, in *model c*, we performed another traditional multiple logistic regression analysis, excluding variables such as birth weight and 5-minute Apgar, which could be affected by smoking and could therefore, be on the causal path between SDP and psychopathology in the offspring. If these variables were mediators, adjusting for them could underestimate the effect of smoking. In this model, we included demographic (the birth year of the children, maternal age, parity, birth order, and maternal psychiatric diagnosis) and socioeconomic variables (e.g., parental relationship status and household income) that could be considered as a common cause of both maternal SDP and later use of psychotropic medication by the children/adolescents. These models (*a, b, c*) were intentionally performed by applying the traditional logistic regression analysis, which do not take into consideration the dependence between siblings within mothers.

Applying a sibling design (*model d*), we next analyzed siblings with discrepant use of psychotropic medication. We compared siblings using a conditional logistic regression analysis with the mother as the grouping variable. In the final *model e,* we expanded *model d* and included birth year of the children, birth order, maternal age, relationship status of the parents, and household income since these variables may change between pregnancies and can be related to both maternal SDP and later use of psychotropic medication by the children/adolescents. The conditional logistic regression is a suitable method to perform sibling analysis [24], because it takes into account the correlation of the information between siblings within mothers, and therefore provides proper adjustment and estimations of the standard errors.

For all analyses, we used Stata version 12 (StatCorp LP. 2011. College Station, TX). We repeated the analyses in “R” version 2.15.1.

## Results

### Characteristics of the Population

Table 1 indicates the characteristics of the offspring population by maternal SDP and use of psychotropic medication in 2005–2008 for both the full and sibling samples. Almost every fourth person in our study population (n = 142,379) had been exposed to maternal SDP, and these children were using psychotropic medication more frequently than those not exposed to maternal SDP. However, while the exposure to maternal SDP was similar for girls and boys, the consumption of psychotropic medication was more frequent in girls.

The use of psychotropic medication increased with age. Smoking during pregnancy declined between 1987 and 1993, meaning that older children in this study population had also been more often exposed to SDP more often. Children born to women <20 years of age had the highest psychotropic drug use, and these young mothers were also the ones most often smoking during pregnancy. It seems that women who had delivered more than three children were more likely to smoke during pregnancy, and their children had higher use of psychotropic medication later on.

Children of women with a psychiatric diagnosis in inpatient registers were at least twice as likely to be using psychotropic medication as children born to women without such diagnoses. Women with a psychiatric diagnosis also smoked during pregnancy almost twice as often as other women. Psychotropic drug use was higher among children whose parents were not living together and in children with low family income. The same factors were also associated with SDP.

Table 2 shows the relation between obstetric characteristics, maternal SDP, and offspring use of psychotropic medication. Birth weight, GA, and birth weight adjusted for GA were correlated in a similar way to both SDP and psychotropic drug use in the offspring. These factors, however, could be on the causal path between SDP and offspring use of psychotropic medication. Apgar score was weakly related to SDP, but individuals with a low 5-minute Apgar had higher use of psychotropic medication.

Compared to the full sample, the sibling sample was rather similar across all characteristics observed in table 1 and 2. However, because the sibling sample was selected for contrast of outcome (cases and control), the proportion of psychotropic use is, as expected, substantially higher in comparison to the full sample. Moreover, because psychotropic use is highly associated to SDP, the prevalence of SDP in the sibling sample is also higher than in the full sample.

### Association between Maternal Smoking during Pregnancy and Offspring use of Psychotropic Medication

**Figure 2:**
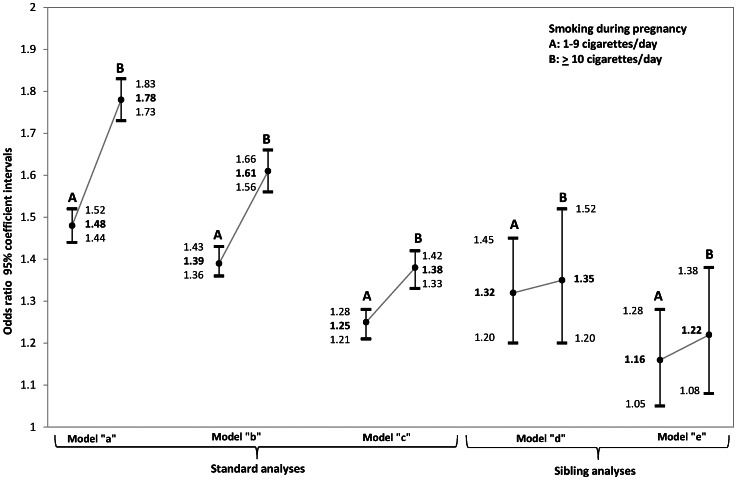
Odds ratios (ORs) and 95% confidence intervals (CIs) showing the association between maternal smoking during pregnancy (SDP) and psychotropic drug use during the period 2005–2008 in children and adolescents born in Sweden between 1987 and 1993. The values were obtained by (a) unadjusted logistic regression analysis, (b) logistic regression adjusted for sex, gestational age (GA), birth weight, 5-minute Apgar score, maternal age, parity, and maternal psychiatric diagnosis, (c) logistic regression adjusted for year of birth, maternal age, parity, maternal psychiatric disease, parents’ relationship status, and socioeconomic variables (household income and whether the parents were receiving social welfare), (d) unadjusted conditional logistic regression stratified by the mother, and (e) conditional logistic regression stratified by the mother and adjusted for birth year, income, and parental relationship status.

Figure 2 shows the results of the logistic regression models. In the crude analysis (model a), the OR for using psychotropic medication was 1.48 for exposure to 1–9 cigarettes and 1.78 for those exposed to ≥10 cigarettes, compared to no smoking.

When we adjusted for the same variables as used by Ekblad et al in their study [5] (i.e., sex, GA, birth weight, 5-minute Apgar score, maternal age, and maternal psychiatric diagnosis) in model b, the ORs were 1.39 and 1.61 for 1–9, and ≥10 cigarettes, respectively.

In the third analysis (model c), we adjusted for a different set of variables than the ones used by Ekblad et al [5]. We excluded variables that we did not consider as confounders (i.e., sex, GA, birth weight, 5-minute Apgar score), and included birth year and socioeconomic variables. The ORs in this model were 1.25 and 1.38 for 1–9 and ≥10 cigarettes, respectively.

In the final part of our analysis (models d and e in Figure 2) we matched cases with control siblings. After adjusting for potential temporal confounders (i.e., birth year, birth order, household income, and parental relationship status at the time of birth), the OR dropped to 1.16 and 1.22, respectively, for 1–9 cigarettes and ≥10 cigarettes.

## Discussion

Applying a conventional multiple logistic regression and similar covariates, we were able to replicate almost exactly the findings recently published by Ekblad et al [5]. According to these results, maternal SDP increases the probability, in offspring, of using psychotropic medication from childhood until young adulthood. However, a stricter sibling analysis accounting for unknown genetic and socioeconomic characteristics of the mothers as well as for observed temporal confounding considerably reduced this association. Our results are analogous to other sibling design studies showing that the relationship between SDP and psychiatric or cognitive outcomes is mainly due to unaccounted familial confounding [12], [13], [14], [15], [16], [17].

We also considered that the probability of smoking in one pregnancy may be conditioned by the experience of the mother in her previous pregnancy, we performed sensitivity analyses to explore whether the order of the exposure between siblings may influences our results. We replicated our sibling analyses in two sub-samples of mothers who had two siblings. The first subsample contained mothers who quit smoking in the second child (n = 1,840) and, the second subsample, mothers who start smoking in the second offspring (n = 1,054). The analyses show the order of the exposure has effect on the association between SDP and psychotropic use in adolescents. The adjusted model for mothers who quit smoking in the subsequent pregnancy shows an OR of 1.70 (0.62; 4.65) and 1.52 (0.54; 4.27) for 1–9 and >10 cigarettes, respectively, while the model for mothers who start smoking indicated smaller effects (ORs 1.07 (0.35; 3.29) and 1.30 (0.40; 4.23) for 1–9 and >10 cigarettes, respectively). These results are very imprecise but they suggest the existence of confounding rather than a causal effect of smoking. In fact, mothers might modify their tobacco habits (e.g., quit smoking) as consequence of health related issued that are a common causes of both changing smoking habits and early determinants of adolescent use of psychotropic drugs. This situation could explain the stronger association found in mother that quit smoking.

The negative effect of smoking on reproductive health outcomes is unquestionable and preventing SDP is of major public health relevance. However, SDP in itself appears to have less impact on offspring psychotropic drug use than suggested by previous conventional analysis. We, therefore, conclude that the results obtained by Ekblad et al [5] were in part due to residual confounding through environmental circumstances in these families or through an inherited risk for psychiatric morbidity. These findings suggest that there are common causes for SDP and use of psychotropic medication. Consequently, further research is needed to identify those factors in order to target appropriate public health interventions.

Our findings are relevant from a public health perspective, because they suggest that a preventive strategy that focuses on the smoking habits of mothers will have little influence on the mental health of children and young adults than previously believe. Rather, there seems to be a need for more complex strategies, with support for socioeconomically weak families and parents with psychiatric disease.

We performed a sibling design, which is a better approach for analyzing causal associations than previous conventional analysis [24]. Moreover, we were able to control for variables which vary between pregnancies such as birth order and maternal age.

Our database contained information from registries covering the entire Swedish population. Moreover, the information in the Swedish Medical Birth Register, the National Patient Register, the National Cause of Death Register, and the Swedish Prescribed Drug Register is reported by law, and the quality of the registries is regularly evaluated by Statistics Sweden and the National Board of Health and Welfare.

However, our study also has some limitations. Sibling comparisons are effective in accounting for unobserved familial characteristics but it cannot rule out any unmeasured confounding factor (i.e., common causes of both maternal SDP and use of psychotropic medication) that simultaneously varies between siblings [23] Furthermore, in spite of the sibling design and the adjustment for observed temporal confounding, we cannot fully exclude the presence of residual temporal confounding. For example, siblings were only matched in terms of the fact that they shared the same mother, but we had no information about the fathers, meaning that some of the siblings might be half siblings which increases differences between sibling by not sharing part of their genetic background. In this sense, it may be possible that the association between maternal SDP and psychotropic drug use in the offspring is confounded by psychiatric morbidity in the father, influencing the smoking pattern in the mother during pregnancy, and being inherited by their offspring.

Another possible source of bias could operate throughout a misclassification on maternal smoking information. Maternal smoking is self-reported by the mother at the first prenatal visit, therefore the information might not be reliable. In this line, a previous study concluded that about 6% of mothers who state they did not smoke during pregnancy were actually smoking [29]. We used information on smoking during early pregnancy as an approximation for SDP, but we did not have reliable information on smoking during late pregnancy. It is, therefore, possible that the pregnant women either stopped or started to smoke after the first antenatal care visit.

A further difficulty is that matching cases to control siblings made it possible to reduce unknown and unaccounted confounding, but it also reduced the number of study subjects and, thereby, the uncertainty of the estimates.

### Conclusions

Our study was able to replicate almost exactly the association between maternal SDP and use of psychotropic medication in offspring, recently reported in Finland by Ekblad et al [5]. However, a more rigorous sibling analysis, which considered unaccounted socioeconomic and familial characteristics and controlled for temporal confounding, reduced this association. Our results are analogous to other sibling design studies concluding that the relationship between maternal SDP and psychiatric or cognitive outcomes observed in conventional analyses is largely due to familial unaccounted residual confounding [12], [13], [14], [15], [16], [17]. Our findings also support the conclusion of Lavigne et al questioning the role of maternal SDP as a risk factor for psychopathology in children [6].

The harmful effect of SDP on reproductive health outcomes is indubitable and preventing SDP is of major public health importance. However, SDP per se appears to have less influence on offspring psychotropic drug use than previous conventional analyses suggest. Identifying the modifiable factors that are associated with both maternal SDP and offspring mental health, and launching public health interventions aimed at these factors seems more relevant than interventions exclusively targeted to maternal SDP in order to address psychiatric morbidity in children and young adults.
